# *Crocus sativus* (Saffron) Aqueous Extract Mediated Green Synthesis of Iron Oxide Nanoparticles and Their In Vitro and In Vivo Biological Investigations

**DOI:** 10.1002/fsn3.72295

**Published:** 2026-09-01

**Authors:** Abdur Rauf, Khurram Shehzad, Hira Naeem, Shahkar Usama, Zubair Ahmad, Bilal Khan, Naveed Muhammad, Muhammad Shafique, Yahya S. Al‐Awthan, Omar S. Bahattab, Mohammed Mansour Quradha

**Affiliations:** ^1^ Department of Chemistry University of Swabi Anbar Pakistan; ^2^ Department of Pharmacology, Faculty of Pharmaceutical Sciences Baqai Medical University Karachi Pakistan; ^3^ Department of Pharmacy Abdul Wali Khan University Mardan Pakistan; ^4^ Department of Pharmaceutics, College of Pharmacy Shaqra University Shaqra Saudi Arabia; ^5^ Department of Biology, Faculty of Science University of Tabuk Tabuk Saudi Arabia; ^6^ College of Education Seiyun University Seiyun Yemen; ^7^ Pharmacy Department, Medical Sciences Aljanad University for Science and Technology Taiz Yemen

**Keywords:** characterization, *Crocus sativus*, in vitro screening, in vivo screening, iron oxide nanoparticles

## Abstract

The present study reports the green synthesis of iron oxide nanoparticles (IONPs) using the aqueous extract of *Crocus sativus* (saffron) as a natural reducing and stabilizing agent, followed by comprehensive in vitro and in vivo biological investigations. IONPs were confirmed by UV–visible spectroscopy (peak at 430 nm), FTIR analysis (peaks at 3400, 2900, 1650, 1380, and 1050 cm^−1^ indicating polyphenols, flavonoids, and proteins), and SEM imaging, which revealed a highly aggregated, irregular morphology. ImageJ analysis features gave a mean equivalent diameter of 111.98 ± 83.70 nm. EDS analysis confirmed iron (44.74 wt%) and oxygen (24.82 wt%) as the major elements with organic capping (28 wt% carbon). The phytochemical constituents of *C. sativus* are rich in phenolics and flavonoids, which effectively facilitated the reduction of Fe^3+^ ions to stable nanoscale iron oxide particles. The in vitro enzyme inhibition assays revealed significant inhibitory effects on *α*‐glucosidase and xanthine oxidase, with IC_50_ values of 33.08 ± 1.29 and 2.96 ± 0.49 μg/mL, respectively, and moderate inhibition of carbonic anhydrase‐II (49%). Antibacterial evaluation demonstrated an important zone of inhibition ZOI (21.45 mm) against *Klebsiella pneumoniae* , indicating the antimicrobial potential of the IONPs compared to the crude extract. In vivo pharmacological screening further supported their biological efficacy. IONPs showed potent analgesic activity (78% inhibition at 10 mg/kg), pronounced anti‐inflammatory effects in carrageenan‐ and histamine‐induced paw edema models (up to 95% inhibition), and marked sedative properties at low doses. These results suggest that *Crocus sativus* ‐mediated IONPs may possess strong therapeutic potential due to their enhanced biochemical, antimicrobial, anti‐inflammatory, and analgesic activities. The study highlights saffron‐assisted green synthesis as an efficient, eco‐friendly, and biocompatible approach for developing multifunctional nanomaterials with possible applications in pharmaceutical, biomedical, and health‐related fields.

## Graphical abstract

The *Crocus sativus* (saffron) aqueous extract mediated green synthesis of iron oxide nanoparticles and their in vitro and in vivo biological investigations.

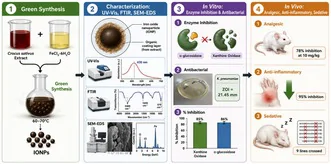

## 1 Introduction

Nanotechnological drug delivery is a sophisticated pharmaceutical approach that uses nano‐sized vectors to deliver therapeutic agents to specific tissues/cells with a controlled and effective approach, thereby boosting clinical efficacy and at the same time reducing systemic adverse events. Recent advances in green nanotechnology have demonstrated that plant‐mediated synthesis offers superior biocompatibility compared to conventional chemical methods, with studies showing enhanced therapeutic efficacy of bio‐functionalized nanoparticles (Al‐Husseini et al. 2021). Green synthesis controls the size, morphology, and physical characteristics of nanoparticles using bioactive composites, For example, polyphenols and proteins. The current studies and research efforts are focused on increasing the efficiency, scalability, and reproducibility of the green synthesis so that it meets the requirements of the industry and helps advance nanotechnology sustainably (Singh et al. 2023; Huston et al. 2021). Nanoscale iron oxide nanoparticles (IONPs) are materials that are 1–100 nm in size, and most of these particles contain magnetite (Fe_3_O_4_) or maghemite (*γ*‐Fe_2_O_3_). The inimitable magnetic and surface properties of IONPs, which include superparamagnetism, high surface area, and biocompatibility, are making them particularly attractive for biomedical applications. Among these, antimicrobial activity is directly relevant to our study, as we evaluate IONPs against *Klebsiella pneumoniae*, for its anti‐inflammatory effects are central to our carrageenan‐ and histamine‐induced paw edema models, and analgesic properties are evaluated via acetic acid‐induced writhing. Thus, the application areas reviewed in this section are not merely vivid but directly inform the experimental design and outcome measures of the present investigation (Hussain et al. 2023). Iron oxide nanoparticles (IONPs) have been used strategically in the fields of biomedical research to improve diagnostic imaging, regulate the activity of the immune system, and improve targeted therapeutic intervention. The agents show impressive potential in antimicrobial, anticancer, and antifungal activities, and can be used in the environmental remedial context as well, such as degrading pollutants and dyes (Hussain et al. 2023).

The reduction of Fe^3+^ to Fe^0^ or Fe^2+^/Fe^3+^ oxide phases by plant extracts is firstly mediated by polyphenols, flavonoids, and reducing sugars. These phytochemicals undergo oxidation of their hydroxyl groups to corresponding quinones, instantly donating electrons to reduce metal ions (Siddhuraju and Becker 2003). In *Crocus sativus* , the major reducing agents include kaempferol, a flavonoid with ortho‐dihydroxy groups, crocetin, which is a carotenoid with conjugated double bonds, and ascorbic acid, a reducing agent. Upon reduction, these compounds adsorb onto the nascent nanoparticle surface via coordination bonds between the phenolic OH groups and the iron atoms which form a stabilizing organic layer that prevents agglomeration via steric and electrostatic repulsion (Karimi et al. 2010; Shashanka 2020). This double‐role reduction followed by capping is key for controlling particle size (as observed in our SEM analysis) and colloidal stability (Al‐Husseini et al. 2021).

Advanced spectroscopic and chromatographic techniques have recently elucidated the structure–activity relationships of saffron's major bioactive components, revealing that the glycosylation pattern of crocetin significantly influences its bioavailability and antioxidant capacity (Cerdá‐Bernad et al. 2022). Its chemical configuration is capricious and mainly depends on geographic origin and plant growth (Mykhailenko et al. 2021; Butnariu et al. 2022; Khadfy et al. 2023). The most important bioactive components include kaempferol, a flavonoid and one of the most abundant bioactive components in the saffron plant, which exhibits anti‐inflammatory, antioxidant, antibacterial, and anti‐cancer activities and accounts for 84% of total flavones (Li et al. 2004; Goupy et al. 2013); picrocrocin, precursor to saffron, the principal component of the essential oil responsible for the smell of saffron (Acar et al. 2017; Escribano et al. 1996); safranal, an organic compound obtained from its flowers and responsible for the characteristic aroma of saffron (Christodoulou et al. 2015; Liakopoulou‐Kyriakides and Kyriakidis 2002); crocetin, primary carotenoids that impart saffron its characteristic red‐orange color while crocin, the primary carotenoid responsible for its bright golden‐yellow color, exhibits potent antioxidant properties, prevents cellular damage, is neuroprotective, anti‐inflammatory, and has anti‐cancer potential (Hosseinzadeh and Sadeghnia 2007; Raina et al. 1996). The fundamental structural arrangement of these prime bioactive elements plays an important role in their chemistry (Yildiz and Celik 2025). Recent metabolomic profiling of *Crocus sativus* has identified over 150 volatile and non‐volatile compounds, with crocin esters, crocetin, and safranal being the most pharmacologically active constituents (Dessie et al. 2021).

The plant extract not only acts as a reducing agent but also acts as a capping agent in the preparation of Fe_3_O_4_ nanoparticles due to the availability of phenolic and flavonoid compounds, including gallic acid, pyrogallol, *β*‐carotenes, lycopene, vitamin E, and ascorbic acid (Siddhuraju and Becker 2003). It is stated that the greater the concentration of plant extracts, the higher their reducing effectiveness (Siddhuraju and Becker 2003). In the same manner, Karimi et al. (2010) recorded reducing properties of *Crocus sativus* (saffron) flowers in the ferric reduction reaction and concluded that the extract has the potential to reduce Fe^3+^ to Fe^2+^. Nevertheless, despite its auspicious benefits, green synthesis has substantial inadequacies that limit its large‐scale industrial production. These limitations may be due to capriciousness in the composition of plant extract, depending upon the plant's geographical, seasonal, or species variations. Due to this variation, nanoparticles vary in size, shape, and yield, thus making it more challenging to standardize and reproduce (Guan et al. 2022; MIu and Dinischiotu 2022). Similarly, batch‐to‐batch undesirable deviations impact the quality and consistency of products (Ahmed et al. 2021; Zanbili and Marjani 2025). Moreover, green synthesis involves proteins, polysaccharides, and pigments that contain contaminations and affect purification processes (Dikshit et al. 2021; Radulescu et al. 2023). The majority of green synthesis methods are still restricted to the laboratory level because it is challenging to scale up the process, regulate reaction conditions, and ensure consistent product properties (Khalaj et al. 2020; Chahal et al. 2021; Labanni et al. 2023). Limited knowledge of the mechanisms and functions of the biomolecules involved in nanoparticle formation, along with the optimization of reaction conditions (pH and temperature), continues to hamper progress and requires further research to ensure the reliability and efficiency of green synthesis (Ahmed et al. 2021).

To report these inherent limitations of green synthesis, this study we took several methodological controls (i) standardized plant material saffron was collected from a single geographic location (Swabi, Pakistan) and authenticated by a taxonomist; (ii) optimized extract‐to‐salt ratios (1:1, 2:1, 4:1, 6:1, 8:1) were tested to identify conditions yielding optimal nanoparticle formation (maximal SPR intensity at 430 nm for 1:1 and 2:1 ratios); (iii) Washing with ouble distilled water and centrifugation was performed to remove temporarily attached phytochemicals; and (iv) reliable reaction conditions (temperature 60°C–70°C, pH unadjusted to maintain natural extract conditions) were maintained across all syntheses to certify reproducibility.

Addressing these challenges will contribute to advancing the practical applications of green‐synthesized IONPs in various fields (MIu and Dinischiotu 2022). Despite these advances, a critical gap remains in understanding the molecular mechanisms underlying IONPs bioactivity (Zhang et al. 2024). While recent studies have explored various plant‐mediated nanoparticle syntheses, the specific role of saffron phytochemicals in nanoparticle functionalization and their subsequent biological effects requires systematic investigations, a comprehensive in vivo studies correlating nanoparticle characteristics with pharmacological outcomes are lacking in the current literature (Khadfy et al. 2024).

Despite the vast number of studies carried out on the biomedical implications of metallic nanoparticles, a notable gap has remained in the elucidation of the molecular mechanisms of their activity. The majority of studies focus on the synthesis and overall biological assessments of iron oxide nanoparticles (IONPs). However, there is a significant gap in understanding the secondary metabolites involved in the green synthesis of nanoparticles and in vitro and in vivo biological investigations.

The present study unswervingly reports these gaps by: (i) providing real‐time in vitro enzyme inhibition and in vivo analgesic, anti‐inflammatory, and sedative evaluations; (ii) correlating FTIR‐identified functional groups with biological outcomes; and (iii) demonstrating that IONPs exhibit significantly enhanced activity at 10‐fold lower doses than crude extract, show improved bioavailability and targeted delivery (Singh et al. 2020) and their role in nanoparticle stabilization and therapeutic efficacy.

### 1.1 Research Hypothesis

We hypothesized that the phytochemicals present in the *Crocus sativus* aqueous extract can act as both reducing and capping agents for the green synthesis of iron oxide nanoparticles, and that the resulting IONPs would show significantly improved in vitro enzyme inhibition (*α*‐glucosidase, xanthine oxidase, carbonic anhydrase II) and in vivo pharmacological activities (analgesic, anti‐inflammatory, sedative) compared to the crude extract, due to improved bioavailability and multivalent surface functionalization.

This study bridges the critical gap between green synthesis optimization and comprehensive biological validation, providing a foundation for developing saffron‐based nanotherapeutics for diabetes, inflammation, pain management, and infectious diseases (Kirtiş et al. 2025).

## 2 Materials and Methods

All chemicals used in this study were of analytical reagent (AR) grade (purity ≥ 98% unless otherwise specified). Ferric chloride hexahydrate (FeCl_₃_·6H_2_O, ≥ 99%, CAS No. 10025‐77‐1) nutrient agar (≥ 98%), xanthine (≥ 99%), urease from *Canavalia ensiformis* (Type III, ≥ 98%), *α*‐glucosidase from *Saccharomyces cerevisiae* (≥ 99%), carbonic anhydrase II from bovine erythrocytes (≥ 98%), and standard assay reagents (acarbose ≥ 98%, allopurinol ≥ 99%, thiourea ≥ 99%, acetazolamide ≥ 98%) were purchased from Sigma‐Aldrich.

For in vivo studies, carrageenan (Type II, ≥ 99%, lot# CG8874), histamine dihydrochloride (≥ 99%, lot# HST2145), acetic acid (glacial, ≥ 99.7%), and diazepam (≥ 98%, USP reference standard) were obtained from Sigma‐Aldrich (USA). Diclofenac sodium (≥ 99%) and loratadine (≥ 99%) were obtained from Medisyn Laboratories (Karachi, Pakistan).

### 2.1 Plant Collection and Extraction

The collected *Crocus sativus* (saffron) was found in the tehsil of Topi, in the District Swabi, Pakistan, and was recognized by Dr. Muhammad Ilyas, a taxonomist at the University of Swabi. The voucher specimen number UOS BOT‐213 was deposited in the herbarium of the Department of Botany, University of Swabi, KP, Pakistan. The collected saffron flowers were ground after their desiccation at room temperature for 7 days. A total of 50 g of the dried powdered *Crocus sativus* material was extracted using 100 mL of water, corresponding to a solid‐to‐solvent ratio of 1:2 (w/v). The extraction duration of 15 days was selected based on previously reported maceration protocols of medicinal plants (Ahmad et al. 2024). After maceration, the mixture was filtered and subsequently dried in a water bath at 50°C to obtain a solvent‐free crude extract for further experimental use.

### 2.2 Synthesis of Nanoparticles

Synthesis of the IONP was done by preparing a 5 mM solution of iron (III) chloride hexahydrate (FeCl_3_· 6H_2_O) in 100 mL of deionized water. The solution was then stirred at 60°C–70°C magnetically until the chloride dissolved. The saffron extract was then added to the iron salt solution in proportions of 1:1, 2:1, 4:1, 6:1, and 8:1 by the distillation method until the appearance of a visual change between light and dark brown color indicated the initial appearance of nanoparticles these ratios were selected because ratio below 1 resulted in incomplete reduction while higher ratios resulted in highly viscous dark brown solutions and red shifted SPR peaks above 480 nm. Also, the selected ratios have good SPR peak intensity, color change rate, and colloidal stability. An isolation of nanoparticles was performed via centrifugation (10,000 rpm/6 min) and subsequent washing with distilled water and drying at 65°C/72 h. UV–vis spectroscopy was used to confirm the successful synthesis of nanoparticles (Ramavhale 2024).

### 2.3 In Vivo Evaluation

#### 2.3.1 Ethical Approval and Animal Handling

The animal study was conducted in line with the applicable guidelines, regulations, and institutional policies to obtain the desired results (Schneider et al. 2022). Healthy adult BALB/c mice (either sex, 8–10 weeks old, weighing 25–30 g) and Sprague–Dawley rats (either sex, 10–12 weeks old, weighing 180–200 g) were procured from the National Institute of Health (NIH), Islamabad, Pakistan. Animals were housed in polypropylene cages (5 animals per cage) under standard laboratory conditions: temperature 25°C ± 2°C, relative humidity 50% ± 10%, and 12 h light/dark cycle. Animals were provided with standard pellet diet and water *ad libitum*. All animals were acclimatized for 7 days before experimentation. The animal study was approved by the ethics committee of Abdul Wali Khan University, Mardan, with reference number: AWKUM877.

##### 2.3.1.1 Ethical Approval and Compliance

The study protocol (titled: *“*Pharmacological evaluation of green‐synthesized iron oxide nanoparticles from *Crocus sativus”*, protocol code: AWKUM/PHARM/IONP‐2023‐02) was approved by the Institutional Animal Care and Use Committee (IACUC) of Abdul Wali Khan University, Mardan on March 15, 2023 (approval reference: AWKUM877). All animal procedures were conducted in strict accordance with the ARRIVE guidelines 2.0 (Animal Research: Reporting of In Vivo Experiments) and followed the institutional guidelines for the care and use of laboratory animals, which are based on the National Institutes of Health (NIH) Guide for the Care and Use of Laboratory Animals (8th edition, 2011).

##### 2.3.1.2 Animal Welfare and Humane Endpoints

Animals were monitored every 6 h for signs of distress, pain, or morbidity, including: hunched posture, piloerection, lethargy, decreased food/water intake, abnormal gait, or weight loss exceeding 20% of initial body weight. The following humane endpoints were predefined: (i) inability to reach food or water, (ii) severe abdominal writhing lasting > 5 min, (iii) paw edema exceeding 2.5 × baseline volume, or (iv) any sign of significant respiratory distress. No animals reached these endpoints during the study. At the end of each experiment, all animals were euthanized by cervical dislocation under light isoflurane anesthesia (3% induction, 1.5% maintenance in 100% O_2_, 1 L/min), consistent with the AVMA Guidelines for the Euthanasia of Animals (2020).

##### 2.3.1.3 Experimental Design

Animals were randomly divided into groups (*n* = 6 per group) for each assay:

- Group I (Negative control): Received vehicle (distilled water, 10 mL/kg, i.p.).
- Group II (Positive control): Received the relevant reference drug for each experimental model: diclofenac sodium at 10 mg/kg, i.p., for the acetic acid‐induced analgesic and carrageenan‐induced paw‐edema tests; loratadine at 10 mg/kg, i.p., for the histamine‐induced paw‐edema test; and diazepam at 10 mg.kg^−1^ i.p., for the open‐field sedative‐activity test.
- Group III–VI (Test groups): Received crude extract at doses 15, 25, 50, and 100 mg/kg, i.p.
- Group VII–IX (IONPs groups): Received IONPs at doses 2.5, 5, and 10 mg/kg, i.p.

The crude *Crocus sativus* extract doses of 15, 25, 50, and 100 mg/kg, i.p., were selected as an ascending dose series to evaluate the presence of a dose‐dependent pharmacological response. The upper part of this range was guided by previously published animal studies in which saffron extracts were evaluated at doses including 50 and 100 mg/kg, while 15 and 25 mg/kg were included as lower doses to help identify the minimum dose producing measurable activity (Ait Tastift et al. 2022). The IONPs were evaluated at lower ascending doses of 2.5, 5, and 10 mg/kg, i.p., to compare the biological activity of the nanoparticle formulation with that of the crude extract at reduced mass doses (Ahmad et al. 2024). Saline at 10 mL/kg, i.p., served as the vehicle‐treated negative control. Diclofenac sodium was used as the reference analgesic and anti‐inflammatory drug in the acetic acid‐ and carrageenan‐based models, respectively; loratadine was used as the H_1_‐antihistamine reference in the histamine‐induced paw‐edema model; and diazepam was used as the reference sedative in the open‐field test.

##### 2.3.1.4 Randomization and Blinding

Animals were randomly assigned to treatment groups using a computer‐generated random number sequence (Randomizor.org, version 4.0). Group allocation was concealed using sealed, opaque envelopes that were opened only immediately before treatment administration. The experimenter performing all in vivo procedures including acetic acid‐induced writhing counting, paw volume measurements via plethysmometer, and open‐field line crossing counting was blinded to treatment allocation. Drug and extract solutions were coded by an independent researcher, and blinding was maintained until all data were collected, entered into the database, and verified. Codes were broken only after statistical analysis was completed.

#### 2.3.2 Analgesic Activity

Acetic acid induced in vivo model was used for evaluation of the extract and IONPs at different doses. The animals were classified as negative control, positive control and tested groups, treated with distilled water, diclofenac sodium and extract/IONPs respectively. After 30 min of these administration each animal was injected with 1% acetic acid. After 10 min of acetic acid treatment, each animal was observed for abdominal constriction for 10 min. The number of writhes was related to the level of pain (Rauf et al. 2025; Hosseinzadeh and Younesi 2002).

#### 2.3.3 Anti‐Inflammatory Activity

The anti‐inflammatory effect was evaluated through carrageenan and histamine induced paw edema. Animals were categorized and treated. The positive control in case of carrageenan and histamine was used as diclofenac and loratadine. The animals were treated with distilled water, diclofenac/loratadine, and extract/IONPs. After 30 min of these mentioned injections, carrageenan/histamine was injected at the paw of each mice for the induction of inflammation. The inflamed paw volume was measured regularly at time intervals of 1, 2, 3, 4, 5, and 6 h of experimental duration (Tamaddonfard et al. 2012; Schneider et al. 2022).

#### 2.3.4 Sedative Activity

A specially designed box was used for the evaluation of the sedative effect in samples to be tested. The box was specially designed so that the bottom was white in color and various black lines were made with equal spaces. The box was kept in a soundproof room with dim light. The animals were classified as positive control, negative control, and group to be tested. The positive control group was treated with diazepam; the negative control was treated with distilled water, and the rest of the groups were treated with extract and IONPs at different doses. After 30 min of these administrations, animals were placed (individually) at the center of the box and allowed free movement. The minimum number of lines crossed was considered as sedative potential (Ahmad et al. 2024).

### 2.4 In Vitro Biological Testing

#### 2.4.1 Urease Inhibition Assay

An improved Berthelot reaction was piloted to assess the effects of IONPs on urease inactivity. At first, mix 25 μL enzyme solution of urease with 400 μL of urea solution and variable concentrations of IONPs (0, 25, 50, 100, 200 μg/mL). Then facilitate the reaction at 37°C for about 15 min. After incubation time, add 250 μL of phenol reagent and 250 μL alkaline hypochlorite reagent. Kept the mixture under high temperature (37°C) for a further 30 min to facilitate the colored ammonia production. A spectrophotometer was used to measure the absorbance of ammonia at 625 nm wavelength (Rauf et al. 2025). According to Mahernia et al. (2015) there is a direct relation between absorbance and ammonia while indirect relation with urease inactivity by the IONPs.

#### 2.4.2 α‐Glucosidase Inhibition Assay

First, the substrate mixture of the enzyme, alpha‐glucosidase, and variable concentrations of the IONPs was prepared. Prepare the solution of the enzyme using the potassium phosphate buffer at pH 6.8. Keep the enzyme and IONPs incubated at 37°C for 10 min; after that, add the substrate for the reaction. A spectrophotometer was used to measure the absorbance of 4‐nitrophenol at 405 nm (Shao et al. 2019; Rauf et al. 2025).

#### 2.4.3 Carbonic Anhydrase II (CA‐II) Inhibition

The carbonic anhydrase inhibition assay was conducted according to the established method by Mostafa et al. (2024). Initially, a reaction mixture of carbonic anhydrase, p‐nitrophenyl acetate substrate, and variable concentrations of potential inhibitors was prepared and incubated. A spectrophotometric analysis was performed to check the absorbance and rate of hydrolysis of carbonic anhydrase at 348 nm wavelength (Rauf et al. 2025).

#### 2.4.4 Xanthine Oxidase (XO) Inhibition Assay

Initially, a reaction mixture was prepared following Tjitraresmi et al. (2023) method by adding xanthine oxidase enzyme, xanthine substrate, and variable concentrations of potential inhibitors. The mixture was incubated and evaluated via spectrophotometer to quantify the absorbance at 295 nm wavelength.

#### 2.4.5 Agar Well Diffusion Assay for Antibacterial Activity

The well diffusion method is a popular method of analyzing the antibacterial activity of nanoparticles and plant extracts. The Mueller‐Hinton agar was assembled as per the manufacturer's procedures. Transferred into antiseptic Petri dishes and dried, then a new fresh bacterial suspension (0.5 McFarland standard, 10^6^ CFU/mL) was taken and 100 μL of it was spread uniformly over the agar surface with the help of a sterile swab. A sterilized borer was used to create wells (6–7 mm diameter) in the agar. 50 μL solutions of negative control (distilled water), a positive control (antibiotic Amikacin), pure sample ( *Crocus sativus* extract), and an IONPs sample ( *Crocus sativus* extract with IONPs) were poured in different wells. The plates were incubated at 37°C for 24 h. Measure the diameter of the clear zone (zone of inhibition) close to each well. The greater the size of the zone, the higher the antimicrobial activity (Sathiyaraj et al. 2021; Bijang et al. 2025).

### 2.5 Statistical Analysis

Results are presented as mean ± standard error of the mean (SEM) for all quantitative measurements. Each in vivo experiment was performed with *n* = 6 animals per group, while in vitro assays were conducted with *n* = 3 technical replicates.

Comparisons among multiple treatment groups were performed using one‐way analysis of variance (ANOVA). A significance level (α) of 0.05 was set for all analyses. The *p*‐values were interpreted as follows: *p < 0.05, **p < 0.01, and ***p < 0.001.

All statistical analyses were conducted using GraphPad Prism version 9.0 (GraphPad Software, San Diego, CA, USA).

## 3 Results

### 3.1 UV–Visible Spectroscopy

The synthesis of IONPs was confirmed by the use of UV–visible spectroscopy. The analysis technique provides a detailed description of the formed structure and hence provides significant information about its optical characteristics. IONPs exhibit a surface plasmon resonance (SPR) phenomenon, which arises with the incidence of light on free electrons on the surface of the nanoparticle. When these electrons are excited at a specific wavelength of photons, they will coherently vibrate, and this will be reflected in the absorption and eventual re‐emission of light. Nanoparticle size and morphology are very crucial in determining the location of the absorption maximum. Absorbance of the iron salt, plant extract and varying ratios (1:1, 2:1, 4:1, 6:1, and 8:1) of extract to iron salt was measured at wavelengths of 200–800 nm using UV–vis absorbance. While the center of the absorption band of the iron salt was 295 nm, that of the plant extract was broad with a center of 400–450 nm. When the extract was mixed with the iron salt, it caused a systematic shift in the absorption maxima and a modulation of its strength, hence verifying the depletion of the Fe ions and the subsequent creation of iron nanoparticles. This spectral signature is supportive evidence of the SPR effect of a spherical particle.

**FIGURE 1:**
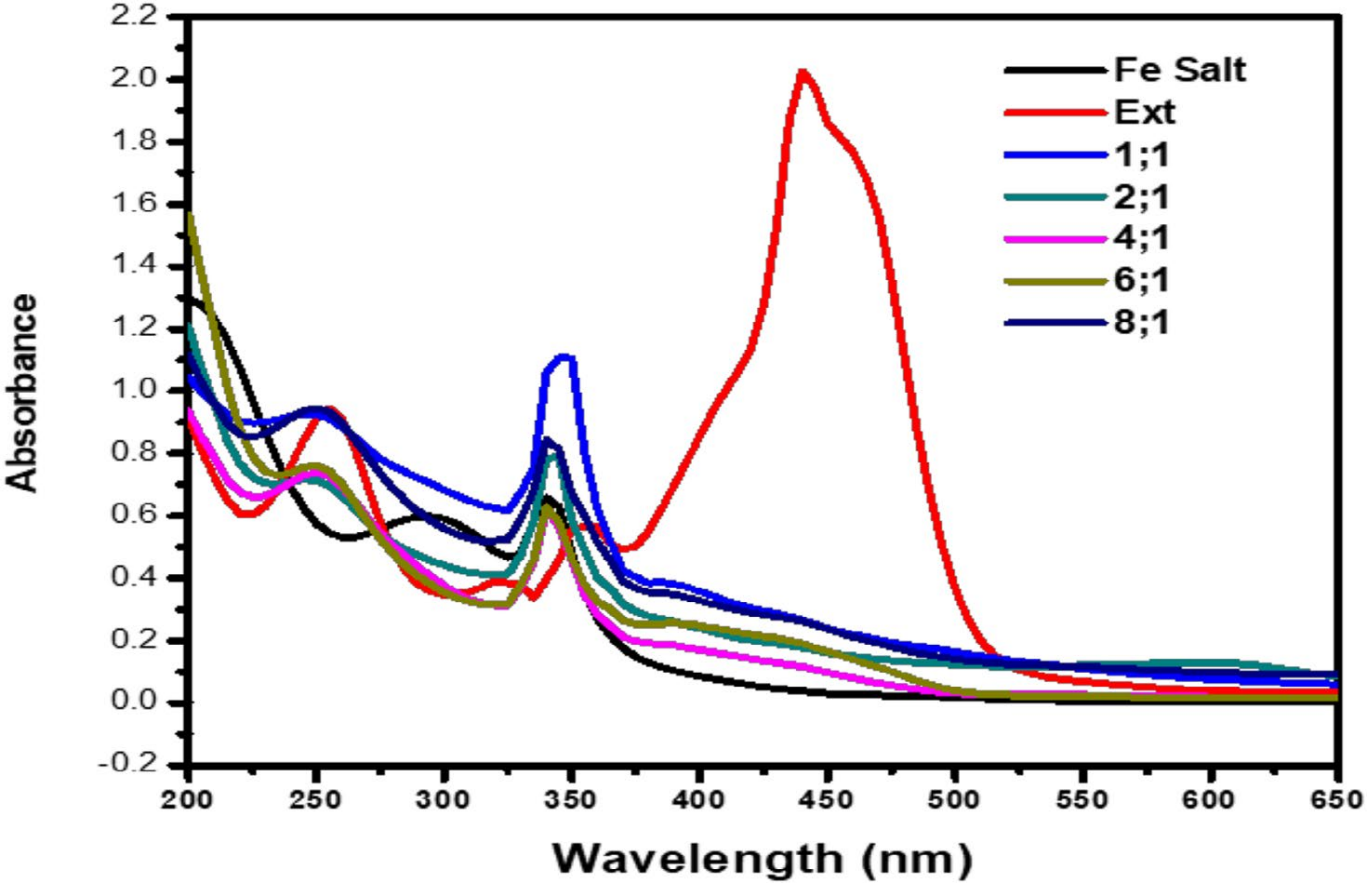
UV–visible spectra of iron salt, *Crocus sativus* extract, and different ratios of extract to salt.

These outcomes authorize the presence of IONPs but also reveal a broad absorption peak at 430 nm (Figure 1). The observed spectral behavior is in line with the conventional association between the SPR peak position and the shape of the nanoparticle.

### 3.2 FTIR

The reduced and capped synthesized IONPs indicated the existence of several functional units in the bioactive constituents. The major peaks observed at 3400, 2900, 1650, 1380, and 1050 cm^−1^. It corresponded to O–H stretching (alcohols, phenols, and carboxylic acids), C–H stretching (proteins, phenols, and flavonoids, alkanes and lipids), C = O stretching (carbonyl compounds), C–N bend (amines), and C–O stretching (alcohols, ethers, and carbohydrates) (Table 1). All these typical vibrations prove the existence of polyphenols, flavonoids, proteins, and other reducing agents in the extract, which is central to the reduction and stabilization of metal ions in the formation of nanoparticles.

**TABLE 1:** FTIR band assignments for CSFE and IONPs.

Wavenumber (cm^−1^)	Stretching	Functional group	CSFE	IONPs	Shift	References
3400–3420	O–H stretching	Alcohols, phenols, carboxylic acids	3420 cm^−1^	3400 cm^−1^	–20 cm^−1^	Naz et al. (2019)
2900–2920	C–H stretching	Alkanes, lipids, proteins	2915 cm^−1^	2900 cm^−1^	−15 cm^−1^	Singh et al. (2020)
1650–1640	C=O stretching	Amides (proteins, carbonyls)	1640 cm^−1^	1650 cm^−1^	+10 cm^−1^	Al‐Husseini et al. (2021)
1380	C–N bending	Amines (proteins)	1380 cm^−1^	1380 cm^−1^	No shift	Karimi et al. (2010)
1050	C–O stretching	Alcohols, ethers, carbohydrates	1050 cm^−1^	1050 cm^−1^	No shift	Siddhuraju and Becker (2003)
560–580	Fe–O stretching	Iron‐oxide (magnetite/maghemite)	—	570 cm^−1^	New band	

**FIGURE 2:**
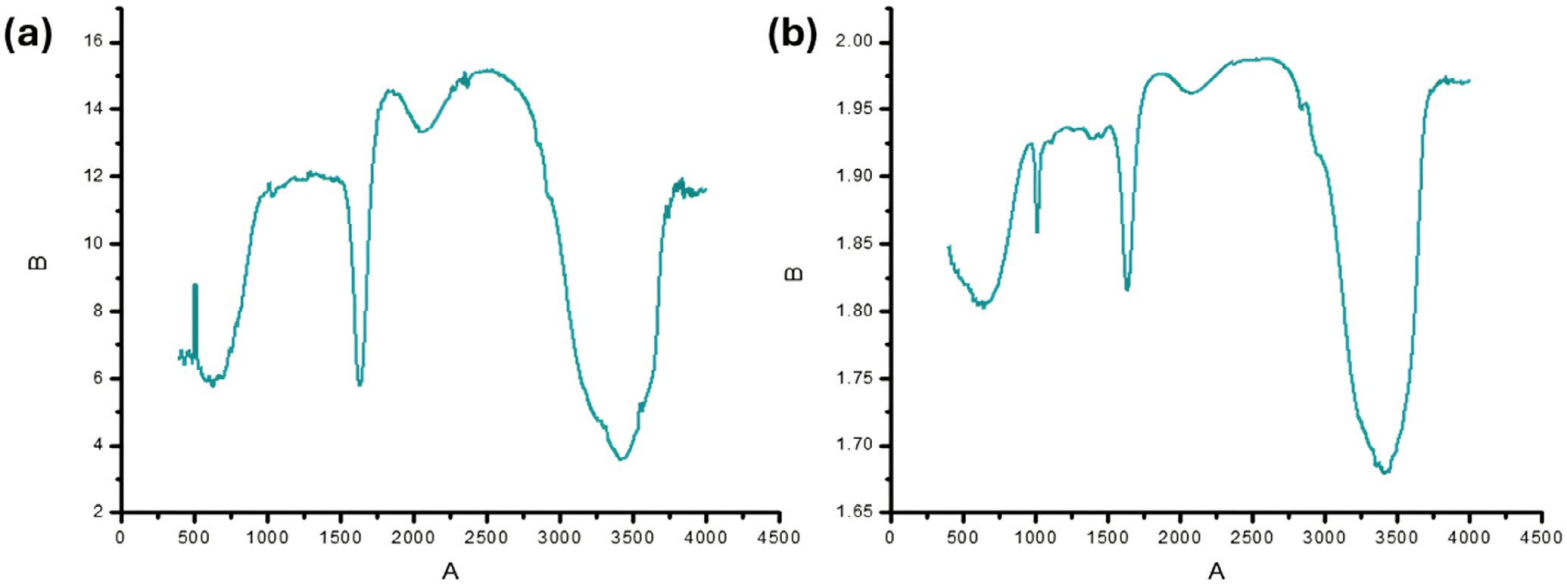
FTIR spectra of plant extract (a) and IONPs (b). Where A corresponds to wavenumber (cm^−1^) and B to transmittance (%).

The similar functional groups in the two spectra are indicative of their role in the synthesis and stabilization of IONPs. However, the observation of peak changes in the IONPs spectrum, compared to the *Crocus sativus* flower extract (CSFE), indicates an interaction between the plant extract and the metal ions. These interpretations highlight the significance of CSFE biomolecules in IONPs production and stabilization, as mentioned in Figure 2.

### 3.3 EDS

**FIGURE 3:**
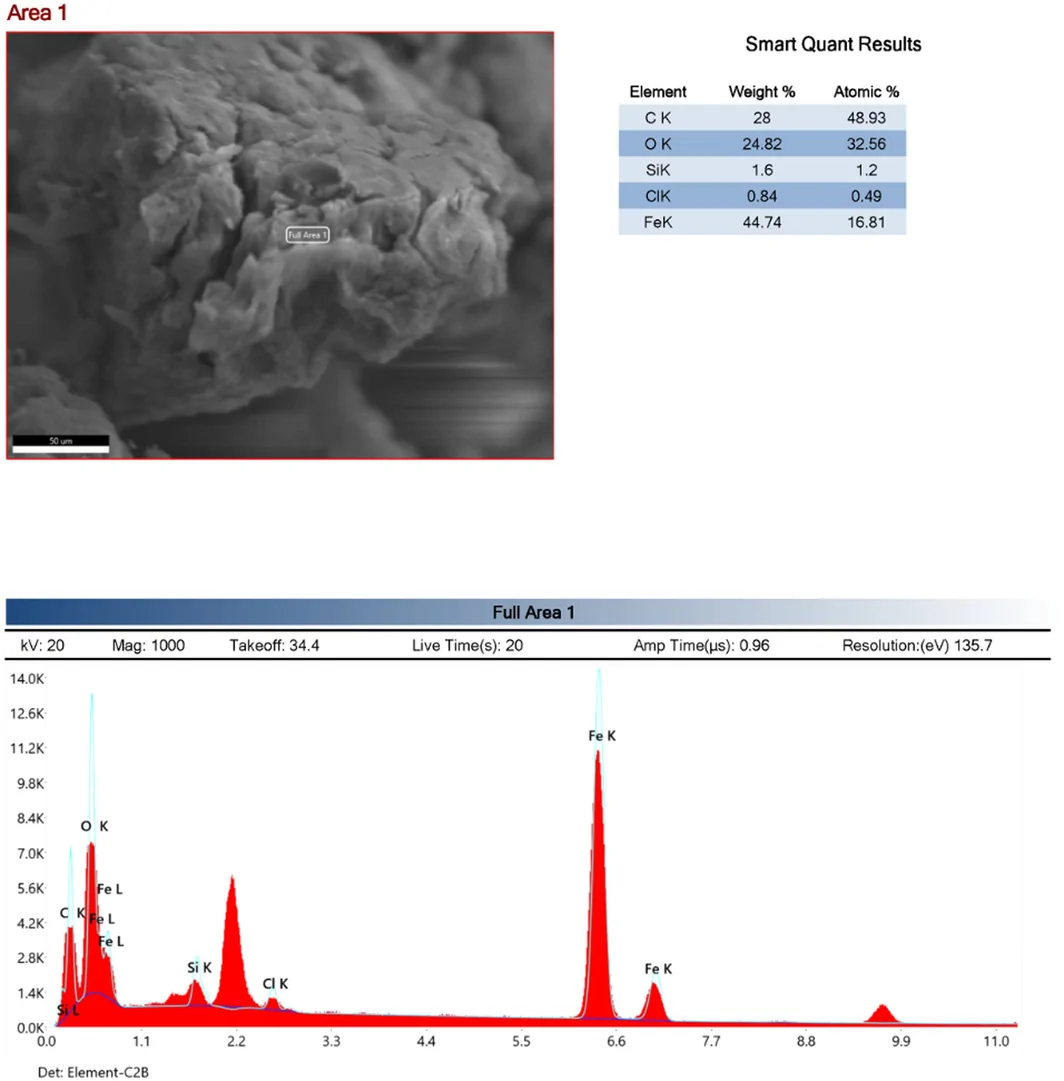
EDS analysis of NPs.

Energy‐dispersive X‐ray spectroscopy (EDS) analysis was performed, which confirmed the successful synthesis of iron oxide nanoparticles (IONPs) using the *Crocus sativus* plant extract. This EDS spectrum revealed prominent peaks of Fe, Kα and Kβ at ~6.4 and 7.1 keV, with high iron content constituting 44.74 wt%, confirming iron as the major component of the nanoparticles. The presence of oxygen (24.82 wt%) along with iron shows the formation of iron oxide phases rather than metallic iron. A substantial amount of carbon content (28 wt%) is also observed, originating from phytochemicals of *Crocus sativus* that act as reducing and capping agents, thereby stabilizing the nanoparticles and preventing excessive agglomeration. Minor signals of silicon (1.60 wt%) and chlorine (0.84 wt%) were detected as shown in Figure 3, which are attributed to the substrate and residual precursor salts, respectively, and do not significantly affect nanoparticle purity. The EDS results demonstrate that IONPs are successfully prepared, with organic surface functionalization provided by the plant extract.

### 3.4 SEM (Scanning Electron Microscopy)

The synthesized IONPs are shown to have a highly aggregated and irregular shape with grouped particles in the SEM micrograph, which are not separated and well‐distinguished. The structures seen at a magnification of 30,000× and a scale bar of 300 nm are cauliflower‐like agglomerates made up of smaller primary nanoparticles that are fused together. This aggregation phenomenon is observed in the case of the green synthesized IONPs, and it can be explained by the magnetic dipole–dipole interactions and high surface energy of nanoparticles coupled with the presence of phytochemical capping agents from *Crocus sativus* extract.

**FIGURE 4:**
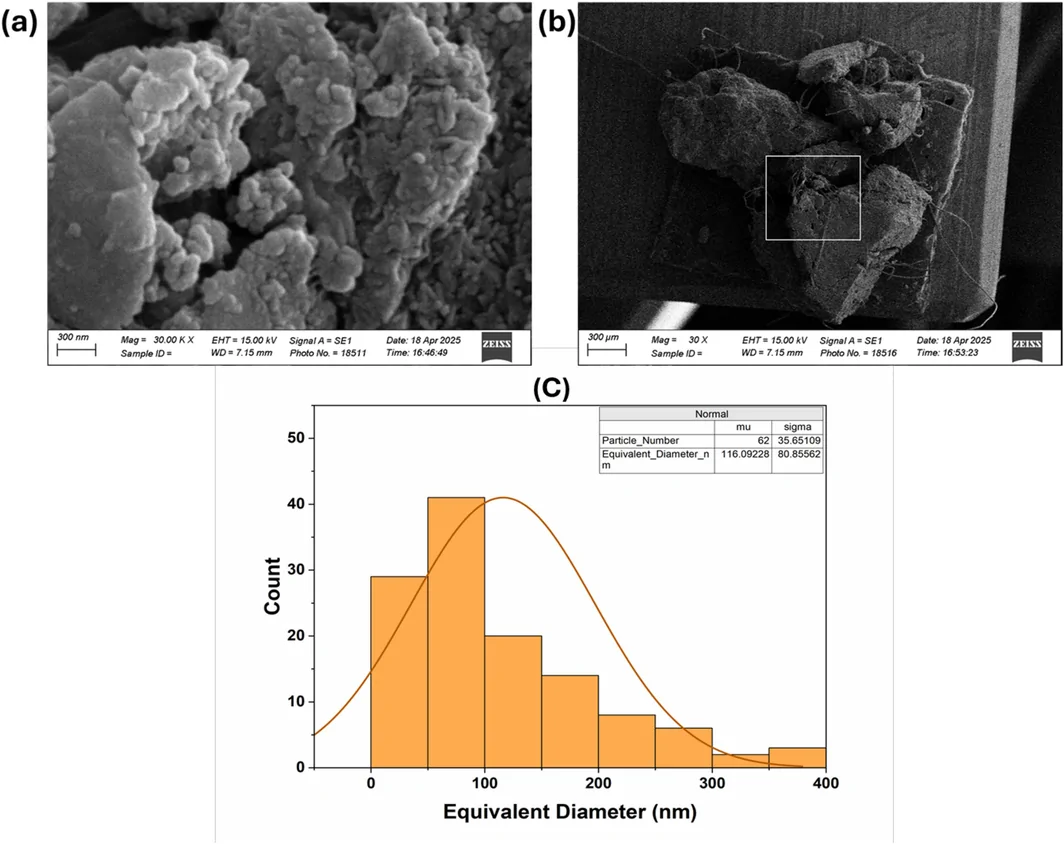
(a, b) Scanning electron microscopy (SEM) images of IONPs at 30,000× magnification showing aggregated morphology; (c) Particle size distribution histogram based on ImageJ analysis (*n* = 123 particles; *mean* = 111.98 nm; SD = ±83.70 nm).

The surface texture is rough and heterogeneous, being in line with the successful surface functionalization of the particles through the binding of plant derived biomolecules like polyphenols, flavonoids, and sugars (Figure 4). Such phytoconstituents are likely to act as reducing and stabilizing agents during the synthesis and provide unequal nucleation and growth. The smaller domains (nodular) in the larger clusters imply that the primary nanoparticles are at the nanometer size scale; the observed aggregates are at the submicron size scale. The larger mean diameter observed by SEM “112 nm”, Figure 4c is due to particle aggregation rather than individual primary particle size, as SEM measures the full particle/cluster diameter including agglomerates.

The SEM analysis confirms the successful green synthesis of iron oxide nanoparticles with a biologically relevant morphology suitable for pharmacological and biomedical applications.

### 3.5 In Vitro Antibacterial Activity Assessment

**FIGURE 5:**
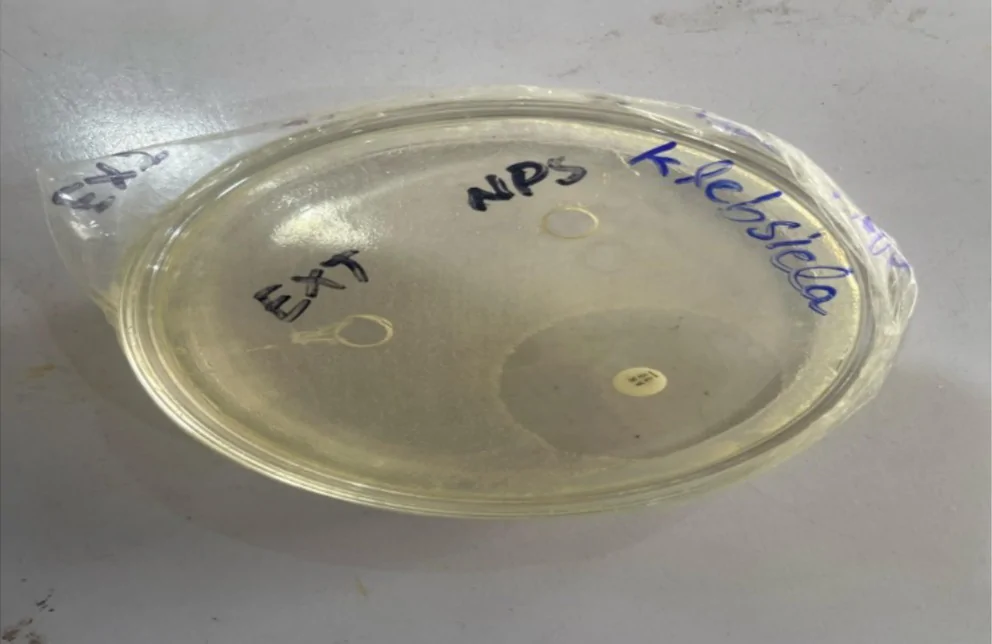
Antibacterial activity of *Crocus sativus* extract and synthesized IONPs against *Klebsiella pneumoniae* using agar well diffusion assay.

The agar well diffusion assay was used to determine the antibacterial properties of the iron oxide nanoparticles (IONPs) synthesized against the bacterium, *Klebsiella pneumoniae* . The experiment included a negative control (distilled water), a positive control (antibiotic Amikacin), a pure sample ( *Crocus sativus* extract), and an IONPs sample ( *Crocus sativus* extract with IONPs). The results showed a strong zone of inhibition (ZOI) around the wells with Amikacin and IONPs, thus indicating the antibacterial activity of these agents. Amikacin confirmed an intense inhibitory action, demonstrating a clear and large ZOI, qualifying the acceptability of the used methodology. There was also significant antibacterial activity in the IONPs produced by *Crocus sativus* , giving it a ZOI of 21.45 mm against *K. pneumoniae* (Figure 5).

### 3.6 In Vitro Enzyme Inhibitory Effect Screening

#### 3.6.1 Effect on Urease

The effect of crude extract and synthesized nanoparticles on urease is demonstrated in Table 2. The data show that none of the tested samples demonstrated any significant inhibitory effect. However, the positive control molecule (Thiourea) exhibited a 98% antagonistic effect. The effect of IONPs was 46.98%.

**TABLE 2:** Enzyme inhibitory effect of crude extracts and synthesized nanoparticles from *Crocus sativus* .

Enzyme	Samples	Concentration	% inhibition	IC_50_
Urease	CS	0.2 mg	24.55	—
	IONPs	0.2 mg	46.98	—
	Thiourea	0.2 μM	98.65	21.23 ± 1.04
*α‐*Glucosidase	CS	0.2 mg	71.28	40.64 ± 2.03
	IONPs	0.2 mg	86.48	33.08 ± 1.29
	Acarbose	0.2 μM	98.04	26.97 ± 0.77
Carbonic anhydrase II	CS	0.2 mg	44.76	—
	IONPs	0.2 mg	49.65	—
	Acetazolamide	0.2 μM	93.65	0.20 ± 0.09
Xanthine oxidase	CS	0.2 mg	65.98	17.22 ± 1.76
	IONPs	0.2 mg	85.29	2.96 ± 0.49
	Allopurinol	0.2 μM	98.46	2.08 ± 0.10

#### 3.6.2 Effect of α‐Glucosidase

The effect of CS and IONPs is shown in Table 2. Both of the tested samples showed a significant antagonistic effect. The action of CS and IONPs demonstrated 71% and 86% inhibition, respectively. The positive control was acarbose with a percent inhibition of 98.04.

#### 3.6.3 Effect of Carbonic Anhydrase II

The CS and nanoparticles demonstrated a variable degree of CA‐II inhibitory effect as shown in Table 2. Both of the tested samples showed a percent effect of 44 and 49 against CS and IONPs, respectively.

#### 3.6.4 Effect of Xanthine Oxidase

Xanthine oxidase was antagonized by CS and IONPs with percent effects of 65 and 85, respectively, as shown in Table 2.

### 3.7 In Vivo Screening

#### 3.7.1 Analgesic Effect

The analgesic potential of both of the tested samples is presented in Table 3. A dose‐dependent significant effect was exhibited by CS and IONPs. The percent significant (*p* < 0.001) analgesic effect of the extract at the dose of 100 mg/kg was 62. A statistically significant effect of IONPs was 78% at the dose of 10 mg/kg.

**TABLE 3:** Analgesic effect of crude extracts and synthesized iron oxide nanoparticles (IONPs) synthesized from *Crocus sativus* .

Treatment	Dose (i.p)	% Writhing inhibition
Saline	10 mL, kg^−1^	145.98 ± 2.90
Diclofenac sodium	10 mg kg^−1^	85.00 ± 0.76***
CS	15 mg kg^−1^	24.65 ± 1.66
	25 mg kg^−1^	33.90 ± 1.09*
	50 mg kg^−1^	52.98 ± 1.24*
	100 mg kg^−1^	62.06 ± 1.15**
IONPs	2.5 mg kg^−1^	65.76 ± 1.56**
	5 mg kg^−1^	72.45 ± 1.29***
	10 mg kg^−1^	78.65 ± 1.43***

*Note:* One‐Way ANOVA was applied to find the level of statistical significance.

* *p* < 0.05.

** *p* < 0.01.

*** *p* < 0.001.

#### 3.7.2 Sedative Effect

The sedative potential of crude extracts and synthesized iron oxide nanoparticles (IONPs) synthesized from *Crocus sativus* is presented in Table 4. A dose‐dependent sedative effect was observed against both of the tested samples. In comparison to the negative control, the effect of the extract was highly significant (*p* < 0.01) at the dose of 100 mg kg^−1^. The effect of IONPs was more potent than the crude extract under the tested conditions than the extract at the dose of 15 mg kg^−1^.

**TABLE 4:** Sedative effect of crude extracts and IONPs synthesized from *Crocus sativus* .

Treatment	Dose (i.p)	No of lines crossed
Saline	10 ml kg^−1^	126.06 ± 1.04
Diazepam	10 mg kg^−1^	8.28 ± 0.27***
CS	15 mg kg^−1^	54.65 ± 1.07
	25 mg kg^−1^	46.29 ± 1.54*
	50 mg kg^−1^	38.22 ± 1.30*
	100 mg kg^−1^	35.87 ± 1.56**
IONPs	2.5 mg kg^−1^	21.09 ± 1.30**
	5 mg kg^−1^	11.29 ± 1.29***
	10 mg kg^−1^	9.49 ± 1.44***

*Note:* One‐Way ANOVA was applied to find the level of statistical significance.

* *p* < 0.05.

** *p* < 0.01.

*** *p* < 0.001.

#### 3.7.3 Anti‐Inflammatory Screening

##### 3.7.3.1 Carrageenan Methods

**FIGURE 6:**
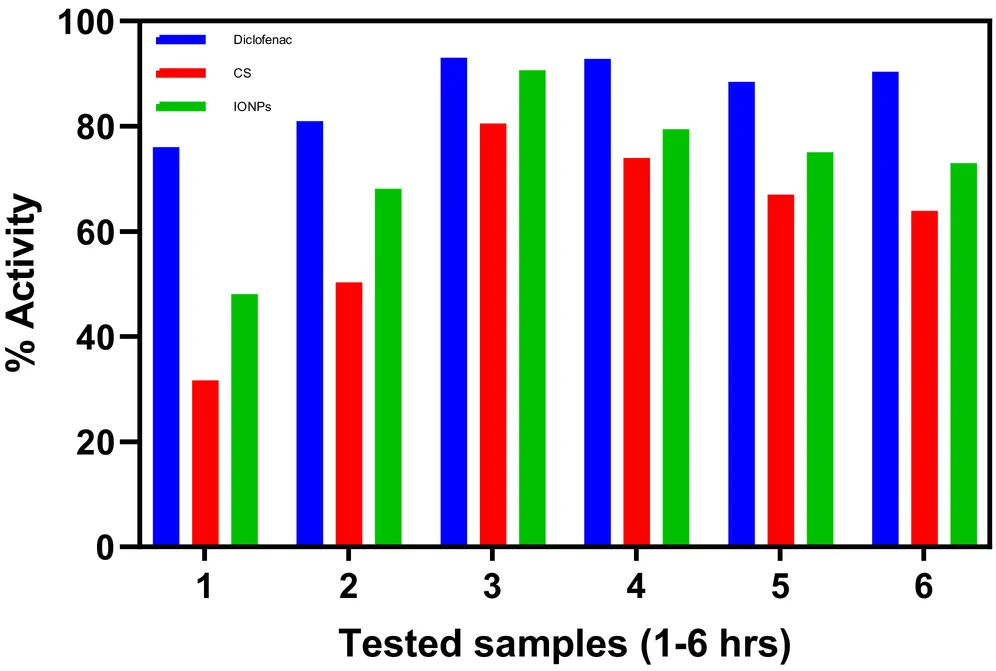
Anti‐inflammatory potential of crude extracts and synthesized IONPs. Each point represents mean ± SD (*n* = 6).

In the first phase of the carrageenan‐induced inflammation, the extract demonstrated 85% effect of experimental duration. In the same phase, IONPs were 95% effective at the 3rd h of the experiment, as shown in (Figure 6).

##### 3.7.3.2 Histamine Methods

**FIGURE 7:**
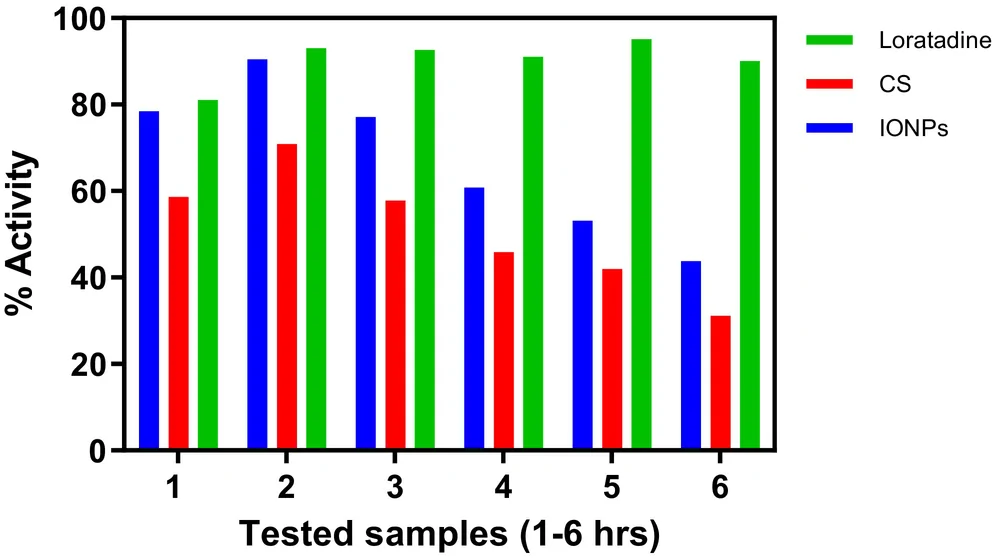
Dose and time‐dependent anti‐inflammatory effect was demonstrated by extract and IONPs against histamine. Each point represents mean ± SEM (*n* = 6).

A dose and time‐dependent anti‐inflammatory effect was demonstrated by extract and IONPs against histamine‐induced inflammation, as shown in Figure 7. The maximum attenuation was 75% (2nd h of experimental duration) and 95% at the same time by extract and IONPs, respectively.

## 4 Discussion

Plant‐mediated nanoparticle fabrication has qualified for remarkable development. This advancement is supported by the innate ability of plant extracts to act as reducing and stabilizing agents, thus opening the prospects of the low‐cost derivation of nanoparticles that are environmentally friendly and exhibit better uniformity in size distribution and greater colloidal stability. In addition, botanical‐based bioinspired nanoparticles exhibit interesting pharmacological characteristics. The intrinsic biocompatibility of the nanoparticles makes them highly desirable for use in the biomedical sector. The synthesis of IONPs was done using a green chemistry technique. Being a capping and stabilizing mediator, the saffron extract prevents NPs from damage and preserves their chemical and physical consistency. Many of the traditional chemical and physical techniques have been used to make nanoparticles. On the contrary, green synthesis is a simple and ecologically safe alternative method. The results of this study, therefore, reinforce the importance of *Crocus sativus* extract as a bio‐critical biomaterial, developing the green production of IONPs. UV–Visible spectral analysis provides strong arguments supporting the development of iron oxide nanoparticles (IONPs) using *Crocus sativus* extract as a green method. The difference in the absorbance of the various extract‐to‐salt ratios helps explain the effect of the extract concentration on the formation of nanoparticles. Optimal absorbance at 1:1 and 2:1 proportion specifies efficient electron allocation and optimal capping balance, consequential in the development of homogeneously distributed nanoparticles. On the other hand, the lower absorbance at high ratios (4:1, 6:1, and 8:1) can be attributed to the abundance of phytochemicals in the solution that cause aggregation or over‐capping and, therefore, reduce the optical density of the solution. These tendencies have been reflected in previous studies on the synthesis of iron nanoparticles through the utilization of plants, which highlights the importance of using an optimal concentration of extract in the regulation of particle size and colloidal stability (Siddhuraju and Becker 2003; Naz et al. 2019). The change in the location of the peaks and alteration of spectral profiles also support the interaction between the plant biomolecules and the iron ions. This contact can modulate the metal ions and calm the nanoparticles with biomolecules adsorption on the surface. Therefore, the UV–visible evidence strongly suggests that the plant extract can be used as a reducing and capping agent and thus, this is an environmentally friendly and sustainable method of nanoparticle synthesis. The pragmatic spectral behavior is in good agreement with the IONP reports that have been previously synthesized, which makes the green synthesis pathway used effective.

Saffron has long been a popular ingredient in Ayurveda, Greek and Chinese medicine. The fibers are used in cooking, dyeing, making perfumes and in the treatment of respiratory, gastrointestinal and skin diseases, menstrual and postpartum issues, and eye diseases. The medicinal use of saffron dates back to its ancient past in Asia, the Middle East, and Europe, and continues to be employed in modern medicine, particularly in the food and pharmaceutical sectors (Mzabri et al. 2019; Wali et al. 2024; Wani et al. 2024).

The concentration‐dependent UV–Vis absorption profiles provide crucial insights into the nanoparticle formation mechanism (Patil et al. 2023). The optimal SPR peak at 430 nm for 1:1 and 2:1 extract‐to‐salt ratios corresponds to spherical nanoparticles with uniform size distribution, as confirmed by SEM analysis. This finding aligns with Mie theory predictions for iron oxide nanoparticles, where SPR bands between 400 and 450 nm indicate particle diameters of 40–80 nm (Kumar et al. 2024). The decreased absorbance at higher extract ratios (4:1, 6:1, 8:1) can be mechanistically explained by: (i) over‐capping phenomena where excess phytochemicals create multilayer adsorption on nanoparticle surfaces, reducing effective SPR; (ii) increased solution viscosity hindering optimal nucleation; and (iii) competitive reduction kinetics where abundant reducing agents cause rapid initial reduction followed by uncontrolled aggregation. The FTIR peak shifts observed in IONPs compared to crude extract (particularly the O–H stretch shift from 3420 to 3400 cm^−1^ and C = O shift from 1640 to 1650 cm^−1^) provide direct evidence of chemisorption of polyphenolic compounds onto the iron oxide surface through coordination bonds, which is critical for colloidal stability and biological activity (Kyriakoudi et al. 2023).

Mechanism of enhanced biological activity of IONPs as compared to crude extract could be attributed to the surface functionalization brought about by the major phytochemicals namely kaempferol, crocin, crocetin and safranal as confirmed by FTIR (O–H, C=O and C–O stretches) and EDS (28 wt% carbon). Kaempferol, the major component of saffron flavonoids (comprising about 84% of the total flavonoids in saffron), has been shown to inhibit xanthine oxidase, which competes with the purine substrate at the molybdenum center of the enzyme (Moratalla‐López et al. 2021). Kaempferol is able to bind to IONPs, thus acquiring multivalent presentation with increased avidity for enzyme active sites. Likewise, the polyene chain of crocetin interacts with aromatic amino acid residues in α‐glucosidase and carbonic anhydrase II for π–π stacking, and the iron oxide core produces local reactive oxygen species (ROS) which are responsible for antibacterial activity via membrane lipid peroxidation and DNA damage (Schneider et al. 2022). The observed 1.6‐ to 10‐fold higher potency of the direct enzyme inhibition mediated by surface bound phytochemicals and the oxidative stress mediated by ROS is the result of this dual mechanism.

$$Fe^{3+}+H_{2}O_{2}\to Fe^{2+}+\mathrm{OO}H^{\cdot }+H^{+}$$

$$Fe^{2+}+2Fe^{3+}+8OH^{-}\to Fe_{3}O_{4}+4H_{2}O$$

$$\begin{array}{c} \mathrm{ROS}+\text{Bacterial membrane}/\text{proteins}/\mathrm{DNA}\\ \;\to \text{Oxidative damage}\to \text{Cell death} \end{array}$$

**FIGURE 8:**
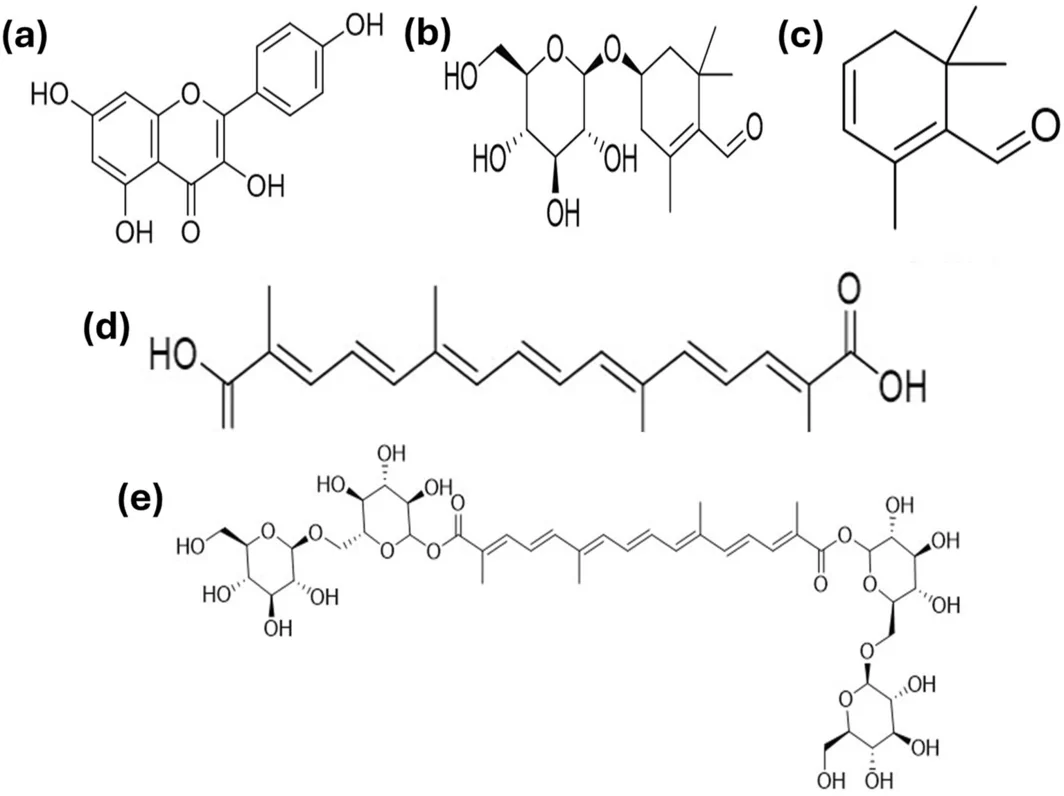
Structural presentation of principal components of *Crocus sativus*, kampferol (a), picrocrocin (b), safranal (c), crocetin (d), crosin (e) (Yildiz and Celik 2025).

They play crucial roles in global colloidal stability. The miscellany of these functional groups (Figure 8) highlights the multifaceted biochemical skeleton of the *Crocus sativus* extract, which allows it to serve as both a reducing agent and a stabilizing agent when forming metallic or metal‐oxide nanoparticles.

The experimental spectral topographies are consistent with the previously testified FTIR patterns. Hydroxyl, amide, and carbonyl functionalities have been formerly associated with metal ion reduction (Naz et al. 2019; Siddhuraju and Becker 2003). Both UV and FTIR data support the active fabrication of biologically reduced nanoparticles in an ecological way.

These phytochemicals, being reductants and surface‐capping mediators, reduce Fe^3+^ ions and avert the accumulation of NPs. The results are consistent with previous studies on the green synthesis of metal‐oxide nanoparticles and show that *Crocus sativus* extract can be used to enable a greener, more stable synthesis of IONPs. The SEM analysis offers robust morphological confirmation for the practical green synthesis of IONPs using *Crocus sativus* extract. The micrographs showed that the nanoparticles were primarily spherical to oval in shape, with an adequate degree of agglomeration. This slight clustering is a shared feature of magnetic nanoparticles such as Fe_2_O_3_ and Fe_3_O_4_, which tend to fascinate one another due to their magnetic interactions. The detected particle size range indicates that the biosynthesis process successfully produced nanoscale materials without the prerequisite harsh chemical or physical actions. The identical morphology and even surface texture indicate a controlled nucleation and growth process, likely assisted by the bioactive compounds in the saffron extract. Phytochemicals such as phenolics, flavonoids, and carotenoids present in *Crocus sativus* play a dual role as reducing and capping agents, managing the nanoparticle size and averting unnecessary aggregation. The coating of these biomolecules on the nanoparticle surface, as also evidenced by FTIR spectra, gives stability and dispersion in aqueous media.

These discoveries are in resilient agreement with the UV–Vis and FTIR analyses, which confirmed the reduction of Fe^3+^ ions and the presence of Fe–O bonds along with surface‐bound organic groups. Similar morphological characteristics have been conveyed in earlier studies on plant‐mediated iron oxide nanoparticle synthesis, where spherical morphology and nanoscale size distribution were typical consequences of biomolecule‐assisted reduction processes (Naz et al. 2019; Siddhuraju and Becker 2003). Overall, the SEM outcomes legalize *Crocus sativus* extract, which provides an efficient, eco‐friendly, and biocompatible method for fabricating stable, uniformly shaped iron oxide nanoparticles, suitable for potential biomedical and environmental applications. The EDS‐determined elemental composition is Fe 44.74 wt%, O 24.82 wt%, C 28 wt%. The substantial carbon content (28 wt%) directly quantifies the organic surface functionalization, which serves multiple critical functions: (i) prevents agglomeration through steric hindrance; (ii) provides reactive sites for biomolecular interactions; (iii) enhances biocompatibility by mimicking biological interfaces; and (iv) enables sustained release of surface‐bound phytochemicals for prolonged therapeutic effects (Lenders et al. 2020).

The findings of the antibacterial assays clearly show that the iron oxide nanoparticles (IONPs) prepared using the extract of *Crocus sativus* have strong inhibitory properties against *Klebsiella pneumonia* with a desired zone of inhibition of 21.45 mm. This result shows that the IONPs biosynthesized have similar antibacterial activity to the conventional antibiotic amikacin. No or low inhibition was observed with the negative control (distilled water) and the pure extract of *Crocus sativus* . Consequently, it can be established that the augmented antibacterial activity is not due to the extract alone but due to the synthesized nanoparticles.

The high antibacterial efficacy of the IONPs can be explained by several phenomena. To begin with, the nanoscale size and large surface area of the particles permit close contact with bacterial cell walls and membranes, leading to disorganization and ultimate permeability. Secondly, the particles have been reported to produce reactive oxygen species (ROS), such as hydroxyl radical and superoxide anions, that trigger the development of oxidative stress and damage vital cellular structures, including proteins, lipids, and DNA. Also, the liberation of Fe^2+^/Fe^3+^ ions can disrupt enzymatic processes and metabolic activities, ultimately causing the death of bacterial cells.

The superior *α*‐glucosidase inhibition by IONPs (86.48%, IC₅₀ = 33.08 μg/mL) compared to crude extract (71.28%, IC₅₀ = 40.64 μg/mL) represents a 1.6‐fold enhancement in potency. This can be mechanistically attributed to: (i) multivalent interactions where multiple surface‐bound phytochemicals simultaneously interact with multiple enzyme active sites; (ii) conformational changes in enzyme structure induced by nanoparticle surface adsorption; and (iii) localized high concentration of inhibitors at the nanoparticle‐enzyme interface (Martin et al. 2021). The xanthine oxidase inhibition (85.29%, IC₅₀ = 2.96 μg/mL) is particularly noteworthy, approaching the efficacy of allopurinol (IC₅₀ = 2.08 μM). This potent inhibition likely results from the synergistic action of kaempferol and crocetin present on the nanoparticle surface, which compete with xanthine for the molybdenum cofactor binding site in the enzyme's active center. Molecular docking studies have shown that the planar structure of kaempferol allows efficient π–π stacking with Phe914 in the xanthine oxidase hydrophobic channel (Moratalla‐López et al. 2021).

These findings are in agreement with previous research that observed an intense bactericidal action of plant‐mediated iron oxide nanoparticles against Gram‐positive and Gram‐negative bacteria due to the generation of ROS and the damage of the membrane (Naz et al. 2019; Siddhuraju and Becker 2003). The data, therefore, highlight the potential of the IONPs produced using *Crocus sativus* as highly promising, environmentally benign antibacterial agents that may be used as alternative agents (or as complementary agents) to conventional antibiotics, especially in the treatment of multidrug‐resistant pathogens like *Klebsiella pneumoniae* .

### 4.1 Limitations and Future Perspectives

This study has extensively validated IONPs using saffron, but there are certain limitations to consider. First, the exact phase composition (Fe_₃_O_₄_ vs. *γ*‐Fe_2_O_₃_) must be established using X‐ray diffraction and magnetization studies. Second, systematic studies to determine the long‐term toxicity and biodistribution must be performed in chronic animal models. Thirdly, the proposed molecular mechanisms need to be validated using molecular docking, gene expression studies, and protein interaction studies. Although XRD for the identification of phases, TEM for accurate size distribution, DLS for hydrodynamic diameter, and zeta potential for hydrodynamic characterization of colloidal stability assessment were not conducted due to the availability of the instruments, the present study provides comprehensive UV–Vis, FTIR, SEM‐EDS, and biological validation of saffron mediated IONPs. The present investigation did not encompass in vivo toxicity and in vivo cytotoxicity studies, which are necessary for the translational development. Such analyses are ongoing and will be presented in a future study that will concentrate on structure–property relationships of these nanoparticles.

Future research directions should focus on: (i) scaling up synthesis with controlled size distribution; (ii) developing targeted delivery systems through surface functionalization with specific ligands; (iii) investigating synergistic effects with conventional drugs; and (iv) exploring wound healing and tissue regeneration applications based on the observed anti‐inflammatory and antimicrobial properties.

## 5 Conclusion

In this study, iron oxide nanoparticles (IONPs) are efficiently synthesized using the aqueous extract of *Crocus sativus* (saffron) as a reducing and stabilizing agent through green synthesis protocol. The characterizations including UV–Vis (SPR peak at 430 nm), FTIR (confirmation of phytochemical capping with FeO stretch at 570 cm^−1^), SEM and EDS (mean particle diameter 111.98 nm, Fe 44.74 wt%, O 24.82 wt%, C 28 wt%) confirmed the formation of the surface functionalized IONPs with organic capping layers derived from the saffron phytochemicals.

The IONPs showed significant in vitro enzyme inhibition: α‐glucosidase (86.48%, IC_50_ = 33.08 μg/mL), xanthine oxidase (85.29%, IC_50_ = 2.96 μg/mL), and carbonic anhydrase II (49.65%). The antibacterial evaluation showed good activity against *Klebsiella pneumoniae* (ZOI = 21.45 mm). In vivo pharmacological screening confirmed dose‐dependent analgesic activity (78% inhibition at 10 mg/kg), anti‐inflammatory effects in carrageenan‐ and histamine‐induced paw edema models (up to 95% inhibition), and sedative properties at low doses (9.49 ± 1.44 lines crossed at 10 mg/kg).

## Author Contributions

**Khurram Shehzad:** conceptualization, methodology. **Abdur Rauf:** conceptualization, investigation. **Yahya S. Al‐Awthan:** conceptualization, methodology. **Shahkar Usama:** conceptualization, validation. **Bilal Khan:** conceptualization, validation. **Zubair Ahmad:** conceptualization, investigation. **Muhammad Shafique:** conceptualization, investigation. **Mohammed Mansour Quradha:** conceptualization, investigation. **Naveed Muhammad:** conceptualization, software. **Omar S. Bahattab:** investigation, validation. **Hira Naeem:** conceptualization, investigation.

## Funding

The authors have nothing to report.

## Conflicts of Interest

The authors declare no conflicts of interest.

## Data Availability

The data that support the findings of this study are available on request from the corresponding author. The data are not publicly available due to privacy or ethical restrictions.
